# Self-reported taste and smell alterations and the liking of oral nutritional supplements with sensory-adapted flavors in cancer patients receiving systemic antitumor treatment

**DOI:** 10.1007/s00520-021-06049-4

**Published:** 2021-02-25

**Authors:** Jacco J. de Haan, Remco J. Renken, Yvette Moshage, Daniëlle A. Kluifhooft, Camille Corbier, Louise E. Daly, Hélène Blanchard, Anna K. L. Reyners

**Affiliations:** 1 Department of Medical Oncology, University Medical Center Groningen, PO Box 30.001, 9700RB Groningen, The Netherlands; 2 Department of Cognitive Neuroscience Center, University Medical Center Groningen, Groningen, The Netherlands; 3 Danone Nutricia Research, Utrecht, The Netherlands

**Keywords:** Taste alterations, Smell alterations, Chemotherapy, Oral nutritional supplements

## Abstract

**Purpose:**

Taste and smell alterations (TAs and SAs) are often reported by patients with cancer receiving systemic antitumor therapy and can negatively impact food intake and quality of life. This study aimed to examine the occurrence of TAs and SAs and investigate the impact of TAs on overall liking of oral nutritional supplements (ONS) with warming and cooling sensations.

**Methods:**

Patients receiving systemic antitumor therapy completed a questionnaire on sensory alterations and evaluated overall liking of 5 prototype flavors of Nutridrink® Compact Protein (hot tropical ginger (HTG), hot mango (HM), cool red fruits (CRF), cool lemon (CL), and neutral (N)) on a 10-point scale via a sip test. Differences between patients with and without TAs were investigated using permutation analysis.

**Results:**

Fifty patients with various cancer types and treatments were included. Thirty patients (60%) reported TAs and 13 (26%) experienced SAs. Three flavors were rated highly with a liking score > 6 (CRF 6.8 ± 1.7; N 6.5 ± 1.9; HTG 6.0 ± 2.0). Larger variation in ONS liking scores was observed in patients with TAs with or without SAs (4.5–6.9 and 4.6–7.2, respectively) vs. patients without TAs (5.9–6.5). TAs were associated with increased liking of CRF (Δ = + 0.9) and N (Δ = + 1.0) flavors.

**Conclusions:**

TAs and SAs are common in patients with cancer undergoing systemic antitumor therapy. Patients with TAs were more discriminant in liking of ONS flavors compared to patients without TAs, and sensory-adapted flavors appeared to be appreciated. The presence of TAs should be considered when developing or selecting ONS for patients with cancer.

**Trial registration:**

Registration at ClinicalTrials.gov (NCT03525236) on 26 April 2018.

**Supplementary Information:**

The online version contains supplementary material available at 10.1007/s00520-021-06049-4.

## Introduction

Taste and smell alterations are often reported by patients with cancer receiving systemic antitumor therapy, with reported prevalences of 56–76% and 16–49%, respectively [1, 2]. The occurrence of taste and smell alterations varies between cancer types and treatment regimens, with many different antitumor agents including irinotecan, taxane-based and platinum-based chemotherapy, and tyrosine kinase inhibitors being associated with high TSA prevalence [2, 3]. In addition, concomitant treatments such as radiotherapy can be important contributing factors.

Taste and smell alterations associated with cancer and its treatment can be variable and have differential impact on patients’ chemosensory capabilities [4]. In patients with cancer, sensory alterations can include hyposensitivity, describing a reduction in taste and/or smell sensitivity, hypersensitivity, evidenced by an increase in taste and/or smell sensitivity, a distortion of normal taste (dysgeusia), or a taste perception without an external stimulus (phantogeusia) [4, 5]. The exact mechanism underlying the diversity in chemosensory profiles observed in patients is not yet fully understood. Sensory alterations have been associated with decreased quality of life in patients with cancer and have a negative impact on social-emotional functioning [6–8].

Taste and smell alterations are considered important causes of malnutrition that is highly prevalent in patients with cancer undergoing systemic antitumor therapy [9–11]. Sensory alterations are associated with a decrease in appetite and a reduction in energy and nutrient intake, adversely affecting patients’ nutritional status and leading to subsequent weight loss [4, 12–15]. Malnutrition is associated with a high morbidity and mortality rate, more complications, and poorer tolerance to antitumor therapy [16, 17]. Therefore, timely assessment of the nutritional state and involvement of the dietitian should become standard practice in the integrated care for patients with cancer. If normal oral intake is not sufficient to meet energy and protein needs, oral nutritional supplements (ONS) are a well-established step to preserve an adequate intake [18].

The effectiveness of ONS for nutrition support depends on the acceptability of the product by the patient and on patient compliance [19]. The presence of taste and smell alterations may impact sensory perception and palatability to ONS [20]. Unfortunately, ensuring compliance to ONS is challenging in daily practice. Therefore, more efforts on the sensory design of ONS are needed to personalize nutritional support, optimize palatability and acceptability of ONS, and to meet the likings of as many patients as possible to support compliance. To compensate for sensory alterations, some patients describe the need for more intense stimuli by deliberately adding more spices, salt, and ginger to their meals to stimulate a sensory response, while others describe a need for less intense flavors [12, 21]. The trigeminal nerve innervates the mucous membranes of the nasal and oral cavities, and plays a fundamental role in chemosensation and the overall “flavors” of food. The trigeminal nerve endings can be activated by an array of physical (mechanical force, temperature) and chemical agents, and can evoke a sensation such as the pungent or sharp feel of chili peppers, horseradish, wasabi roots, and Szechuan pepper, the coolness of peppermint, and the tingle of carbonated drinks [22]. These preferences could be incorporated into the sensory development of ONS, to better address the sensory alterations of patients with cancer.

Usually, explorations of the appreciation of different ONS have been performed in healthy volunteers. Less is known about the likings of patients with sensory alterations who undergo systemic antitumor treatment. This study aimed to explore the occurrence of taste and smell alterations in patients receiving systemic antitumor therapy, and to investigate overall liking of new ONS prototype flavors specifically designed to better address sensory alterations in these patients.

## Materials and methods

### Selection of patients

Patients were selected randomly and approached to participate in this study at the oncology ward or outpatient clinic at the Department of Medical Oncology of the University Medical Center Groningen. All patients were at least 18 years old and were receiving systemic antitumor therapy. Patients were excluded if they had an aversion to milk-based beverages or an aversion to flavors used in the study, including lemon, red fruits, exotic fruit, and yellow fruit. Other exclusion criteria were any coexisting comorbidities affecting taste or smell function, food allergy or intolerance for one of the ingredients used, dysphagia, inability to swallow nutritional drinks, severe comorbidities (renal failure/liver failure/heart failure) or nausea, vomiting, or diarrhea during the last 24 h. The study was approved by the ethical committee of the University Medical Center Groningen, registered at ClinicalTrials.gov (NCT03525236), and reported according to SPIRIT guidelines.

### Composition and flavors of the ONS

Nutridrink® Compact Protein is a low-volume, high-protein, energy-dense ONS. Each 125 ml bottle contains 306 kcal and 18 g of protein. Five prototype ONS flavors, designed for patients with sensory alterations, were evaluated. Four flavors were based on 2 different types of flavor providing a warming (hot tropical ginger (HTG) and hot mango (HM)) or cooling sensation (cool red fruits (CRF) and cool lemon (CL)), and 1 flavor was based on a neutral profile (neutral (N)). Warming flavors (HTG and HM) contain capsicum derivatives and were designed to trigger warming/spicy sensations that activate the trigeminal nerve. Cooling flavors (CRF and CL) contain menthol derivatives and were designed to have a fresh mouthfeel and cooling sensation that activates the trigeminal nerve. The neutral (N) flavor was specifically designed to support patients in times of hypersensitivity to taste and odors by not providing additional stimuli.

### Product tasting and data collection

Patients willing to participate filled out a screening questionnaire, containing questions regarding age, gender, type of cancer, type of treatment, stage of treatment, and current use of ONS. Patients fulfilling all inclusion criteria and giving informed consent continued with a product questionnaire. The product questionnaire was used for the liking scores of the different ONS flavors, containing questions about the overall liking, flavor, sweetness, texture, warming/cooling sensation, and color. The study was performed in a quiet and well-ventilated room between 10AM and 3PM. The test was performed at least 1 h after finishing breakfast or lunch. Patients tasted (sip test, 30 ml) each of the 5 different ONS flavors in a randomized, blinded, sequential-monadic test. Between the tastings, 5-min breaks were provided during which patients drank water and ate unsalted crackers to neutralize their taste. For each product tasted, the participants were asked to answer a questionnaire including 1 question on the overall liking of the product using a 10-point hedonic scale (1 being extremely dislike to 10 being extremely like), with scores ≥ 6 being at least sufficient and scores ≤ 5 being poor. Mouth feeling was also evaluated on this scale. More detailed organoleptic evaluations of the product (including the intensity of the color, sweetness, flavor, and sensation intensity (cooling/warming)) were collected using the 5-point Just About Right (JAR) scale (1, not enough at all; 2, not enough; 3, just about right; 4, too much; 5, far too much). Following the completion of the product questionnaire, patients filled out questions about changes in their taste and smell. Patients were also asked if they experience any other symptoms since starting the treatment from a list of symptoms and were asked to check all that apply. The study-specific questionnaire consisted of questions examining if the sense of taste and smell changed compared to before current antitumor treatment (I do not taste/smell anything at all; I smell/taste less; I don’t perceive taste/smell in the same way I did before; I detect new unpleasant taste/smells; I do not perceive any changes in taste; smell), the severity of taste and smell alterations (none, mild, moderate, severe), the extent to which taste/smell alterations impact on daily life (not at all, a little, quite a lot, very much), and questions specifically the extent of changes in taste intensity to sweet, salty, sour, bitter tastes (much stronger, a little stronger, no change, a little weaker, much weaker).

### Statistics

Statistical analysis was completed using in-house written scripts in MATLAB 2018b (MathWorks, Natick, MA). Descriptive results on overall product liking are reported as mean and standard deviation. Testing for significance was performed using permutation statistics. A null distribution of a given statistic can be obtained by randomly relabeling samples, e.g., randomly assigning a subject to a group.

Differences in overall liking score per flavor between patients with and without taste alterations were obtained by comparing the difference of group mean against a null distribution. The null distribution is obtained by permuting the subject labels 1000 times. That is for each permutation, subjects are randomly assigned to one of the groups and the differences of group mean were calculated. Results are deemed significant if the true difference of group mean falls outside the 95th percentile (α < 0.05, two-sided, uncorrected for multiple comparisons).

Within each group—the group without taste or smell alterations, the group with taste and smell alterations, and the group with taste alterations but without smell alterations—the products were compared against each other (10 comparisons) by calculating the difference in product liking score per subject and subsequent averaging. These values were compared against the null distribution obtained using permutation methods (1000 permutations). Under the null hypothesis, the difference in product liking is expected to be zero. For each permutation, the difference in liking score per subject is flipped (negative becomes positive and vice versa) for a subset of subjects, randomly selected. Subsequently, the group average product difference is obtained for all comparisons. The null distribution is built by retaining the maximum—across comparisons—of the absolute group difference. This way, a multiple comparison correction over the 10 comparisons is obtained. Results were considered significant if the true difference between products fell outside the 95th percent interval (α < 0.05, two-sided), FWE-corrected across 10 comparisons.

## Results

### Patient characteristics

In total, 55 patients were invited to participate in the study, of whom 50 gave informed consent. Baseline demographic and clinical characteristics are displayed in Table 1. Overall, 30 (60%) patients were male and 36 (72%) patients were 55 years or older. Patients with different cancer types were included. Urogenital cancers were most prevalent (26%), followed by colorectal cancer (20%). The intention of the treatment was palliative for most patients (68%) and the majority (68%) was treated with chemotherapy. The median duration since the start of the current treatment was 2 months. Of 50 included patients, 8 patients used ONS daily.

**Table 1:** Demographic and clinical characteristics of oncological patients receiving antitumor medication (*N* = 50).

Variable	N (%)
Gender	
Male	30 (60%)
Age (years)	
18-34	5 (10%)
35-54	9 (18%)
55+	36 (72%)
Current on ONS	
Yes	8 (16%)
Cancer type	
Urogenital cancer	13 (26%)
Colorectal cancer	10 (20%)
Sarcoma	5 (10%)
Esophageal/gastric cancer	4 (8%)
Gynaecological cancer	4 (8%)
Brain tumors	3 (6%)
Breast cancer	3 (6%)
Hepatobiliary cancer	3 (6%)
Melanomas	2 (4%)
Neuroendocrine tumors	2 (4%)
Lung cancer	1 (2%)
Intention of treatment	
Palliative	34 (68%)
Curative	8 (16%)
Neo-adjuvant	5 (10%)
Adjuvant	3 (6%)
Type of treatment	
Chemotherapy	34 (68%)
Chemoradiotherapy	5 (10%)
Chemotherapy with targeted therapy	5 (10%)
Immunotherapy	4 (8%)
Targeted therapy	2 (4%)
Duration of treatment (months)	
< 3	26 (52%)
3-12	19 (38%)
>12	5 (10%)

### Sensory alterations

Thirty patients (60%) reported taste alterations and of those 13 (26%) patients experienced smell alterations. No patients had smell alterations only. Of patients with taste alterations, 13 (26%) reported hypogeusia (12 patients perceived less taste and 1 patient had no taste at all) and 16 (32%) did not perceive tastes the same way as before (dysgeusia). In patients with taste alterations, the severity was reported as moderate-severe by 40% (*n* = 12) with an impact on daily life rated as moderate-severe by 37% (*n* = 11) (Table 2). In patients experiencing taste alterations, the majority of patients reported no change in salty, sweet, sour, or bitter tastes. In total, 9 (30%) and 5 (16%) patients reported a weaker/much weaker taste for salty and sweet flavors, respectively, while 5 (17%) and 3 (10%) reported a stronger/much stronger taste for sweet and sour flavors (Supplementary Table 1). Overall, 21 patients reported experiencing a bad taste, of which chemical (*n* = 12, 57%) and metallic tastes (*n* = 10, 48%) were common. In patients with smell alterations, the severity was reported as moderate-severe in 6 patients (46%), while the impact on daily life was reported to be moderate-severe in 7 patients (54%; Table 3). Next to taste and smell alterations, other symptoms reported by at least 10% of patients are nausea (30%), early satiety (26%), fatigue (26%), no appetite (22%), vomiting (18%), constipation (14%), and mouth sores (10%) (Supplementary Table 2).

**Table 2:** The severity of taste alterations that patients experience and their impact on daily life (*n* = 30)

		Severity of taste alterations					
		None	Mild	Moderate	Severe	Missing	Total
Impact of taste alterations	None	2	5	3	0	0	10 (33%)
	Mild	0	7	1	0	1	9 (30%)
	Moderate	1	2	4	1	0	8 (27%)
	Severe	0	0	0	3	0	3 (10%)
	Missing	0	0	0	0	0	0 (%)
	Total	3 (10%)	14 (47%)	8 (27%)	4 (13%)	1 (3%)	30 (100%)

**Table 3:** The severity of smell alterations that patients experience and their impact on daily life (*n* = 13)

		Severity of smell alterations				
		None	Mild	Moderate	Severe	Total
Impact of smell alterations	None	0	2	1	0	3 (23%)
	Mild	0	2	1	0	3 (23%)
	Moderate	1	2	3	0	6 (46%)
	Severe	0	0	0	1	1 (8%)
	Total	1 (8%)	6 (46%)	5 (38%)	1 (8%)	13 (100%)

### Sensory alterations and product liking

**Fig. 1:**
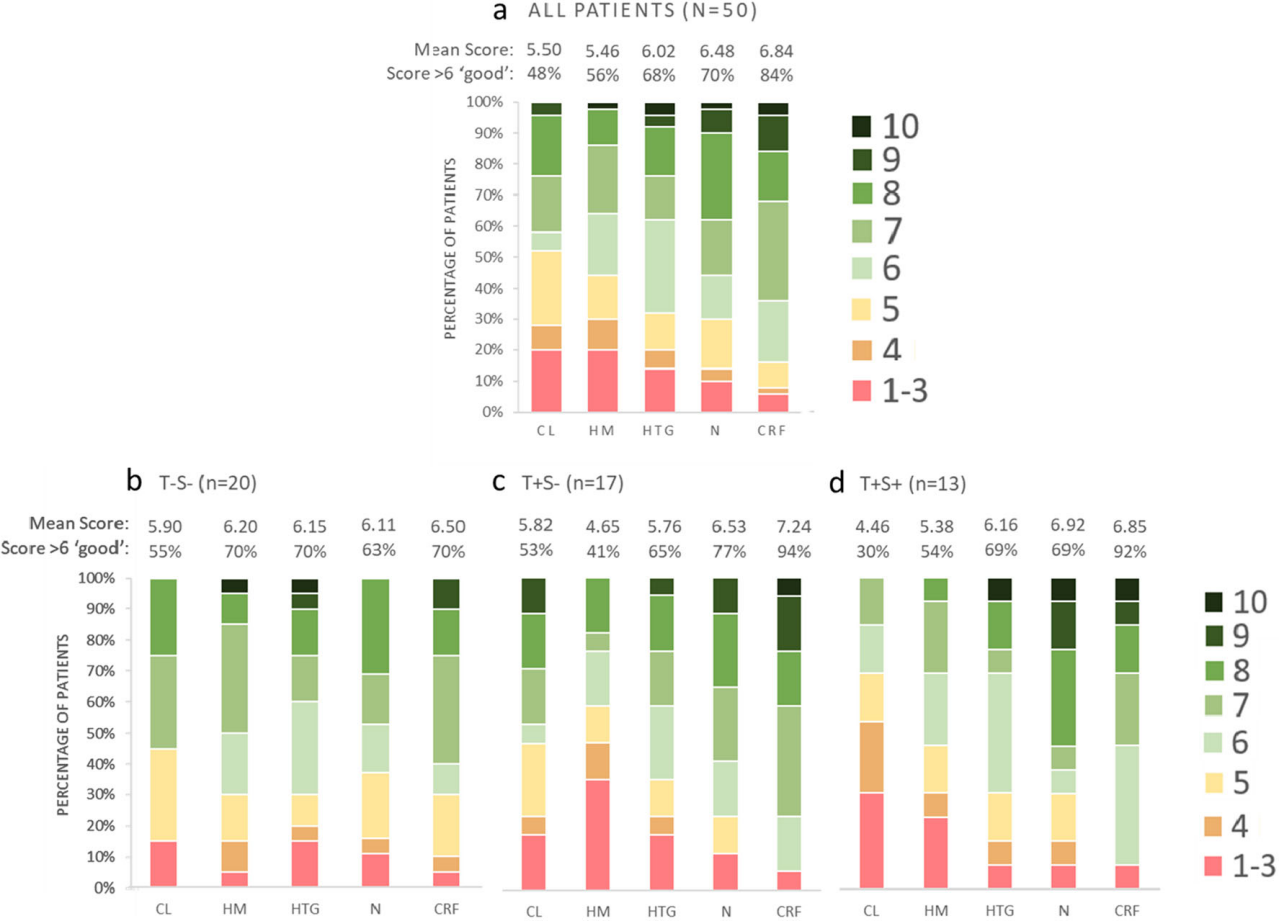
Product liking in all patients and according to the presence of sensory alterations. Product liking in a range from 1 to 10 is given for all patients (**a**), for patients without taste or smell alterations (T-S-) (**b**), for patients with taste alterations without smell alterations (T+S-) (**c**), and for patients with taste and smell alterations (T+S+) (**d**). CL, cool lemon; HM, hot mango; HTG, hot tropical ginger; N, neutral; CRF, cool red fruit

For all 50 patients included, three flavors were rated highly with average liking scores > 6 (cool red fruits (CRF) 6.8 ± 1.7, neutral (N) 6.5 ± 1.9, and hot tropical ginger (HTG) 6.0 ± 2.0), whereas cool lemon (CL) and hot mango (HM) were rated with an average liking score of 5.5 ± 2.3 and 5.5 ± 2.0, respectively (Fig. 1a). Larger variation in overall liking score across the ONS flavors was observed in patients with taste alterations with or without smell alterations (4.5–6.9 and 4.6–7.2, respectively) vs. patients without taste or smell alterations (5.9–6.5; Fig. 1b–d). Of the 5 prototype flavors tested, 3 flavors were rated highly by patients with taste alterations (with/without smell alterations) with liking scores > 6 reported by 92/94% of patients for CRF, 69/77% of patients for N, and 65/69% of patients for HTG.

**Fig. 2:**
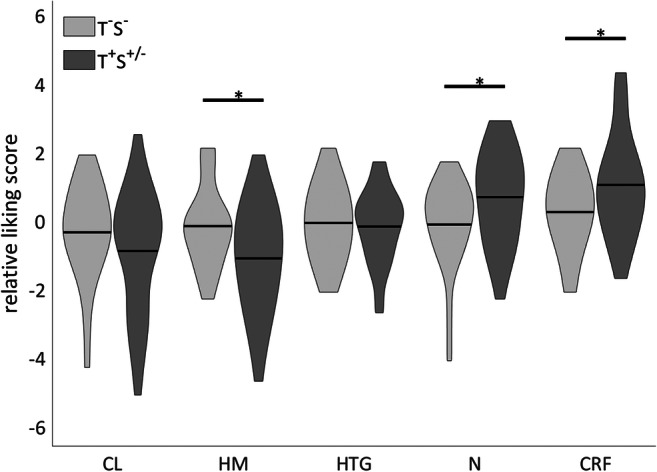
Relative liking score per product for patients with and without taste alterations. CL, cool lemon; HM, hot mango; HTG, hot tropical ginger; N, neutral; CRF, cool red fruit. *p < 0.05

**Fig. 3:**
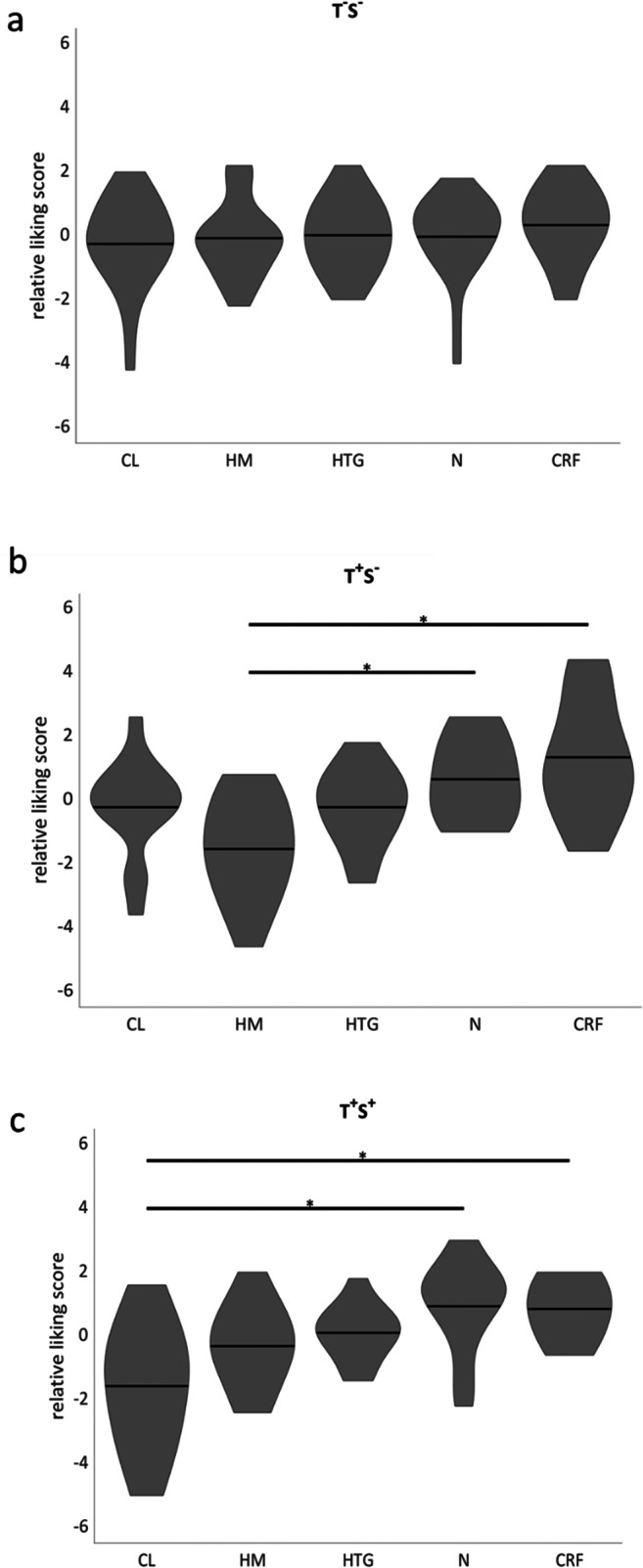
Overall liking of the 5 ONS prototype in **a** patients without taste or smell alterations (T-S-), **b** patients with taste but without smell alterations (T+S-), and **c** in patients with both taste and smell alterations (T+S+). CL, cool lemon; HM, hot mango; HTG, hot tropical ginger; N, neutral; CRF, cool red fruit. *p < 0.05

Post hoc analysis showed that taste alterations were associated with an increase in overall liking for CRF (Δ = 0.9; *P* < 0.05) and neutral (Δ = 1.0; *P* < 0.05) and a decrease in overall liking for HM flavor (Δ = − 1.1; *P* < 0.05; Fig. 2). Next, in each group (no taste or smell alterations, only taste alterations, or both taste and smell alterations), the liking of the ONS flavors was compared. In patients without taste or smell alterations, no differences between the liking of the ONS flavors were observed (Fig. 3a). In contrast, in patients with taste alterations without smell alterations, significantly different liking scores were observed between HM vs. CRF and HM vs. N (both *P* < 0.05; Fig. 3b). Patients with both taste and smell alterations assigned different likings to CL vs. CRF and CL vs. N (both *P* < 0.05; Fig. 3c).

### Flavor intensity and sweetness of the ONS flavors

Overall, the intensity of the ONS flavors was evaluated by most people as “just right” for all products (Supplementary Fig. 1a). A minority considered the flavor intensity to be “much too strong” (highest: HM 16%; and lowest: N 2%), whereas only very few individuals considered the flavor intensity to be “much too weak” (highest: N 6%). With regard to the sweetness of the product, the evaluation “just right” was given by about half of the patients (highest: HTG 52%; and lowest: CL 40%) (Supplementary Fig. 1b). A minority of patients (highest: CL and N 16%; and lowest: HM, HTG, CRF 14%), especially patients without taste alterations, evaluated the ONS flavors as being much too sweet. No significant differences in the reported flavor intensity and sweetness were observed between patients with or without taste alterations.

### Perception of warming and cooling sensations in the ONS

The perceived intensity of the warming sensation in the HM and HTG flavors, and the cooling sensation of the CL and CRF were investigated with the neutral flavor used as negative control.

In terms of the warming sensation, 32% and 34% rated the strength of the warming sensation as “just right” in the HM and HTG flavors, respectively (Supplementary Fig. 2a). The strength of the warming sensation for the HM and HTG was experienced as “much too strong” in 13% and 12% of patients, respectively. No significant differences in the perception of warming sensation were observed between patients with or without taste alterations.

The cooling sensation in the CL was rated as “just right” by 43% of the patients vs. 54% of patients in CRF (Supplementary Fig. 2b). Respectively, 31% and 22% of patients reported the cooling sensation as “much too weak” in the CL and CRF flavors. Almost none of the patients considered the cooling sensation as “much too strong.” No significant differences in the perception of cooling sensation were observed between patients with and without taste alterations.

### Other product characteristics

The mouth feeling was given a score of ≥ 6 out of 10 in 82% of patients for CL, 92% for HM, 84% for HTG, 86% for N, and 88% for CRF. The color was evaluated as just right by the majority of patients (83% of all scores).

## Discussion

The present study assessed the occurrence of taste and smell alterations in patients receiving systemic antitumor therapy and investigated patient likings of ONS with new flavors designed to better address sensory alterations. Overall, patients with taste alterations were shown to be more discriminant in overall liking score per flavor compared to patients without taste alterations. Of the five prototype flavors, 3 sensory-adapted flavors (CRF, N, HTG) were rated positively by most patients.

In this non-select cohort of patients, taste alterations are shown in 60% of patients and smell alterations in 26% of patients, which is in line with previous reports [2, 8, 23, 24]. The clinical consequences of taste and smell alterations highlight the importance of identifying and managing such symptoms [2]. In patients with taste alterations, the impact on daily life was reported to be moderate to severe in 37% of patients. Previous studies have reported that sensory alterations are associated with worse social-emotional function, emotional and role function, physical function, and poor overall quality of life [6, 21]. Sensory alterations in advanced cancer have also been reported to contribute to a substantial decrease in calorie intake of 430–1100 kcal/day, significantly increasing the risk of malnutrition in this patient population [2]. Collectively, these findings highlight the individualized nature of taste and smell alterations among patients with cancer and the adverse impact they can have on daily life and nutritional state.

Nutritional counseling and the prescription of ONS are recommended as first-line nutritional therapies in patients with cancer as ensuring the adequate provision of energy and proteins is the first step in attenuating weight loss and malnutrition [18]. Nutritional intervention studies in patients with cancer have been proven beneficial at improving overall energy and protein intake, overall body weight, muscle mass, and some aspects of quality of life [25–29]. However, the success of ONS at improving nutritional status, patient-reported outcomes, and clinical outcome is dependent on patient compliance to ONS, which can be challenging. Evidence suggests that acceptability and liking of the ONS is an important factor in the compliance to ONS [30, 31]. Within this study, three of the sensory-adapted flavors were appreciated by patients (with average liking scores > 6/10), with many of the sensory attributes rated as “just right” by the majority of patients. A good overall liking of ONS flavors may have potential to support improved compliance; however, this warrants further investigation in longer term clinical studies. Other common barriers to ONS compliance include lack of patient involvement and education, early satiety, and bloating [30, 31]. These factors should be addressed simultaneously to positively impact on compliance to ONS in patients with cancer.

Within this cohort, we report a larger variation in overall liking score per product flavour in patients with taste alterations vs. patients without taste alterations. Previously, in patients with gastrointestinal cancer, more diversity in liking score per ONS product was shown after 6 weeks of chemotherapy compared to the start of treatment. This finding may be a result of changes in taste and smell alterations during treatment [32]. Among patients with taste alterations, the CRF flavor was liked best, and the liking of the CL flavor was lower. This finding indicates that in addition to the fresh and cooling sensation, also the flavor itself has impact on product liking. Therefore, taste alterations should be taken into account in clinical practice when selecting ONS for patients with cancer and also into the sensory development of ONS. Furthermore, these findings highlight that evaluation of acceptability and liking of ONS should be conducted with patients and not healthy subjects, given the impact of taste alterations on product liking. In daily practice, efforts should be made to assess the presence of sensory alterations during systemic antitumor treatment which can impact on the palatability to ONS and a variety of ONS flavors should be offered to patients to identify which flavors are preferred at that time.

It should be noted that this exploratory study was performed in a heterogenous small patient population with large variation in patient and tumor characteristics, and systemic treatments received (chemotherapy, immunotherapy, and targeted therapy). Some patients also underwent radiotherapy or had recently finished another line of systemic antitumor treatment that may have impacted the liking of the products as this may have impacted the severity of taste alterations experienced. Next, large variation in the duration of the present antitumor therapy was observed. As the severity and characteristics of taste alterations change during treatment, further studies are needed to assess taste and smell alterations in patients with cancer over time [9, 33, 34]. It should be noted that only patient-reported TSAs were analyzed and no objective testing for TSAs was performed. In addition, patients tested a few sips of each ONS flavor only, while ONS usually is prescribed for longer periods, at least weeks. Therefore, additional testing is needed to explore the liking of ONS in patients with sensory alterations after prolonged exposure.

In conclusion, this exploratory study confirms that a considerable part of a non-select cohort of patients with cancer undergoing systemic therapy experienced taste and smell alterations. Patients with taste alterations demonstrated a larger variation in overall liking score per product flavor compared to patients without taste alterations. Overall, three of the sensory-adapted flavors were appreciated by patient with cancer, particularly in patients with taste alterations. Therefore, the presence of taste alterations should be considered when developing new flavors, as well as in clinical practice when offering flavor options to patients with cancer.

## Supplementary Information

ESM 1 (DOCX 104 kb)

## Data Availability

The authors had full control over the data and will allow the journal to review our data if requested to do so.
