# Supplementary material for: Self-reported taste and smell alterations and the liking of oral nutritional supplements with sensory-adapted flavors in cancer patients receiving systemic antitumor treatment
Source: Support Care Cancer. 2021 Feb 25;29(10):5691–9. doi: 10.1007/s00520-021-06049-4 (PMC8410716; doi:10.1007/s00520-021-06049-4)
Supplement: Supplementary file 1 — (DOCX 104 kb) [file 520_2021_6049_MOESM1_ESM.docx]

**Supplementary material**

**Supplementary Table 1.** Change in taste of salty, sweet, sour and bitter in patients with taste alterations (n=30). The ‘Missing’ row indicates the number (percentage) of patients who did not answer the specific question.

|  | **Salty** | **Sweet** | **Sour** | **Bitter** |
| --- | --- | --- | --- | --- |
| Much weaker/ I cannot taste at all | 0 (0%) | 1 (3%) | 1 (3%) | 0 (0%) |
| A little weaker | 9 (30%) | 4 (13%) | 1 (3%) | 2 (7%) |
| No change | 16 (54%) | 16 (53%) | 22 (74%) | 24 (80%) |
| A little stronger | 1 (3%) | 4 (14%) | 2 (7%) | 1 (3%) |
| Much stronger | 1 (3%) | 1 (3%) | 1 (3%) | 0 (0%) |
| Missing | 3 (10%) | 4 (14%) | 3 (10%) | 3 (10%) |

**Supplementary Table 2.** Prevalence of symptoms experienced by patients since starting anti-tumor treatment.

| Symptom | N (%) |
| --- | --- |
| Nausea | 15 (30%) |
| Fatigue | 13 (26%) |
| Quickly feeling saturated (satiety) | 13 (26%) |
| No appetite | 11 (22%) |
| Vomiting | 9 (18%) |
| Constipation | 7 (14%) |
| Sores in the mouth | 5 (10%) |
| Swallowing difficulty | 4 (8%) |
| Diarrhoea | 3 (6%) |
| Dry mouth | 1 (2%) |
| Pain | 2 (4%) |

**Supplementary Fig. 1a.** Evaluation of the flavor intensity of the five ONS products in patients with (T^+^S^+^ and T^+^S^-^) and without taste alterations (T^-^S^-^).

**Supplementary Fig. 1b.** Evaluation of the sweetness intensity of the five ONS products in patients with (T^+^S^+^ and T^+^S^-^) and without taste alterations (T^-^S^-^).

**Supplementary Fig. 2a.** Evaluation of the warming sensation of 3 ONS flavors in patients with (T^+^S^+^ and T^+^S^-^) and without taste alterations (T^-^S^-^).

**Supplementary Fig. 2b.** Evaluation of the cooling sensation of 3 ONS flavors in patients with (T^+^S^+^ and T^+^S^-^) and without taste alterations (T^-^S^-^).
